# Video modelling for reducing anxiety related to the use of nasal masks place it for inhalation sedation: a randomised clinical trial

**DOI:** 10.1007/s40368-014-0139-7

**Published:** 2014-11-07

**Authors:** A. Al-Namankany, A. Petrie, P. Ashley

**Affiliations:** 1 College of Dentistry, Taibah University, PO BOX: 2898, Al Madina Al Monawra, 43353 Saudi Arabia; 2 University College London-Eastman Dental Institute, London, UK

**Keywords:** Modelling, Children, Anxiety, Sedation, Nasal mask

## Abstract

**Aim:**

A randomised controlled trial to investigate if video modelling can reduce the level of dental anxiety and increase the patient’s acceptance of the nasal mask usage for children receiving dental treatment using inhalation sedation (IS).

**Methods:**

A sample of 80 (8–16 years) children due to have dental treatments under IS were randomly allocated to either the modelling video or the control video (oral hygiene instruction). The level of anxiety was recorded before and after watching the video on the Abeer Children Dental Anxiety Scale and each child’s ability to cope with the subsequent procedure was assessed on the visual analogue scale. A two-group Chi-square test was used as the basis for the sample size calculation; a significance level of 0.025 was chosen rather than the conventional 0.05 to avoid spurious results arising from multiple testing.

**Results:**

Children in the test group had significantly less anxiety after watching the video than those in the control group throughout the subsequent dental procedure; particullary, at the time of the nasal mask administration (*P* < 0.001).

**Conclusions:**

Video modelling appeared to be effective at reducing dental anxiety and has a significant impact on the acceptance of the nasal mask administration for Inhalation Sedation in children.

## Introduction

Anxiety can be defined as a multi-system response to a supposed threat or danger. It comprises a combination of biochemical changes in the body and aspects of the patient’s personal history, memory and the social situation. Anxiety may occur without cause, or it may be based on a real situation that leads to a reaction that is more than would to what would normally be expected. Severe anxiety can have a serious impact on daily life and affect quality of life and its different effects, such as speaking, eating, and appearance, and through these also social interaction (Luoto et al. 2008).

Dental anxiety is cumulative over time, and its development is influenced by multiple variables. It is most likely to start in childhood (Tickle et al. 2009). It is relatively common in dentistry, affecting approximately 9 % of children in normal populations in Australia, Canada, Europe, and America (Todd and Lader 1991). The British National Children’s Dental Health Survey found that the proportion of children who were dentally anxious steadily increased during the primary school years and then levelled off in secondary school to about 50 % of the population (Todd and Lader 1991). Indeed, several studies reported the strong relationship between dental anxiety and avoidance of dental care (Arnrup et al. 2003). This anxiety is a barrier to carrying out dental treatment safely and simply in the dental chair.

Dental anxiety can act as a major source of stress for general dental practitioners who treat the anxious patient (Girdler and Hill 2009). In that study anxious patients required approximately 20 % more chair time than did low-anxious patients. Moreover, dental anxiety could interfere with the efficiency of treatment by more frequent interruptions. Anxious children demand more resources in terms of time and expertise (Ayer 2005).

Nitrous oxide inhalation sedation (IS) is a weak analgesic offered to children with mild to moderate anxiety to help them to accept dental treatment. It should not be used in isolation from the support given to a child by their dentist. The onset of action is rapid, the effects easily are titrated and reversible, and recovery is rapid and complete. Additionally, nitrous oxide/oxygen inhalation provides a variable degree of analgesia, amnesia, and gag reflex reduction. The need to diagnose and treat, as well as the safety of the patient and practitioner, should be considered before the use of IS (EAPD 2008). The problem of the nasal mask acceptance in children has not been reported in literature but it has been noted in practice.

Modelling is a technique based on psychological principle that people learn about their environment by observing other’s behaviour, using a model, either live or by video. Video modelling is an effective tool for behaviour change; it has been used in medicine, sports and other fields. It is used extensively with autistic children and children with anxiety (Charlop-Christy et al. 2000; Weinstein et al. 2003). Video modelling could be effective for dental anxiety reduction. However, it is not commonly used by general dental practitioners and there is a lack of randomised controlled clinical trials (RCT) in this area (Wright et al. 1991). A similar separate RCT was completed with this current study and it suggested that video modelling appeared to be effective at reducing dental anxiety and has a significant effect on needle phobia in children (Al-Namankany et al. 2014).

To date, there has been no RCT to investigate the effect of video modelling on the behaviour of anxious children receiving dental treatment. Therefore, the objective of this RCT was to investigate the effect of video modelling on the dental anxiety level of those children receiving dental treatment with local analgesia facilitated by IS throughout the procedure and in particular to investigate if video modelling can influence a patient’s anxiety on administration of the nasal mask.

The null hypothesis was that there was no difference between the video modelling and the control groups in the mean dental anxiety scores of participants when video modelling is used to reduce anxiety for children having dental treatment under inhalation sedation.

## Methods

The trial design was a hospital based parallel RCT. The ethical approval for the study was obtained from the Charing Cross Hospital, London, (UK) ethics committee; Reference number: 08/H0711/63. A statistically calculated sample size of 64 children was used. To allow for those not completing the trial, a total of 80 children were recruited, 40 in the test group and 40 in the control group. Two videos were used for this study, the modelling video which showed a dentist applying the nasal mask prior to placing a dental restoration under IS for a 9-year-old girl; and the control oral hygiene instruction video with the same dentist and girl in non-clinical setting. Two information sheets were designed for parents or legal guardians, and a child patient, respectively. Written consent was obtained from parents or those with legal responsibility for the child, and children were also asked for their verbal assent.

The inclusion criteria were: the availability of DVD facilities at home; children aged 8–16 years; healthy children with American Society of Anesthesiologists ASA scale, class I and II; and children who were assessed to be dentally anxious based on the score of ≥26 on the Abeer Children Dental Anxiety Scale (ACDAS) (Al-Namankany et al. 2012). The exclusion criteria were: children who did not meet the inclusion criteria; children with learning disabilities; children who needed emergency dental treatment and children with previous experience with IS. The participants were randomly allocated into intervention (modelling video) and control groups with the aid of computer-generated random numbers by the statistician (AP); these were entered into sealed envelopes that were opened in sequence in accordance with patient participation. All participating children and the dentists providing dental treatment were blinded to the type of video. The chief investigator (AA) randomly allocated the participants to their groups (modelling or control). All participating children were reported as anxious on the ACDAS as a base line measure, and had no previous experience with nasal mask of inhalation sedation.

### Study measures

#### ACDAS

The Abeer Children Dental Anxiety Scale (ACDAS) was used to assess the dental anxiety scores on the first visit as a baseline score prior to watching the video (Al-Namankany et al. 2012). It was also used on the second visit after watching the video and immediately before the start of the dental treatment. It could therefore be used to compare the dental anxiety scores before and after watching the videos.

#### VAS

The visual analogue scale (VAS) was used as a supplementary assessment tool on the second visit during treatment and throughout eight stages of the visit. VAS score was used as it is recommended to use more than one scale in any dental anxiety study (Melamed 1986) and also because it would not have been practical to repeat the ACDAS index at regular intervals throughout treatment, whereas the child could just put a mark on the VAS. The VAS score was determined by measuring in millimetres from the left hand end of a 100-mm line to the point that the patient marked his or her response, where the extreme left side of the line indicated ‘Not afraid at all’ and the extreme right side indicated ‘Very afraid’. The assessment stages were: VAS1, sitting in the waiting area; VAS2, entering the dental clinic; VAS3, sitting on the dental chair; VAS4, dental examination with mirror; VAS5, nasal mask application for IS; VAS6, dental injection; VAS7, tooth drilling; and/or VAS8, tooth extraction.

### Study procedure

The recruitment for the study started from October 2010 and continued until March 2011. The entire procedure was carried out by the chief investigator (AA) who attended the new patients’ clinic daily for 6 months to introduce the project to the target group, gave them the information sheet, obtained the consent, and assessed their anxiety on the ACDAS. The interviews took place in the Department of Paediatric Dentistry and the School of Hygiene and Therapy at the Eastman Dental Hospital UCLH (London, UK). On the first visit, the chief investigator enrolled those children meeting the inclusion criteria to the study and allocated them randomly to either the modelling or control groups. She then gave them the relevant video to watch at home, asked them to arrive 15 min earlier for their second visit and requested that they not tell any dental staff about which video they had watched.

On the second visit, the chief investigator met each of the participants and displayed the relevant video to each child on the computer. She then asked the child to report his/her anxiety on the ACDAS, and the parents or legal guardian to complete the feedback questionnaire immediately before the child entered the dental clinic. The child also reported his/her dental anxiety on the VAS throughout the dental treatment visit.

### Data analysis

A histogram was drawn to show the distribution of age by gender and groups (test/control). The mean, standard deviation (SD), and the 95 % confidence interval (CI) of the mean were evaluated for age in each video group. The total scores for the dental part of the ACDAS can range from 13 to 39 with a cut-off point of ≥26 to indicate anxiety. The baseline ACDAS score in the first visit, before watching the video, for all participants was ≥26. The total ACDAS was measured for each patient at each visit, and the difference in the scores between the first and the second visit was measured by subtracting the score of Visit 2 from the score of Visit 1. The mean, SD, and the 95 % CI of the mean were measured for the difference of the ACDAS total for test and control groups, and the difference was plotted on a histogram for each group. In order to compare the mean difference from the first to second visit in the total ACDAS dental anxiety score between the test and control groups, a two-sample *t* test was used.

A two-sample *t* test was used in order to compare the VAS score (expressed as a percentage) between test and control groups for each of the clinical stages, The mean, SD, and the 95 % CI of the mean were evaluated for the difference of the VAS for test and control groups, and the difference was plotted on a histogram for each group.

## Results

### Demographic results

In order to assess the participant’s eligibility, 174 children (8–16 years) were approached by the chief investigator and 94 children were excluded on the first visit for the following reasons:

- The child was referred to GA (91.5 %, *n* = 86)
- The child refused to participate (1.1 %, *n* = 1)
- The mother refused to participate (2.1 %, *n* = 2)
- Did not met the inclusion criteria (5.3 %, *n* = 5).

**Fig. 1:**
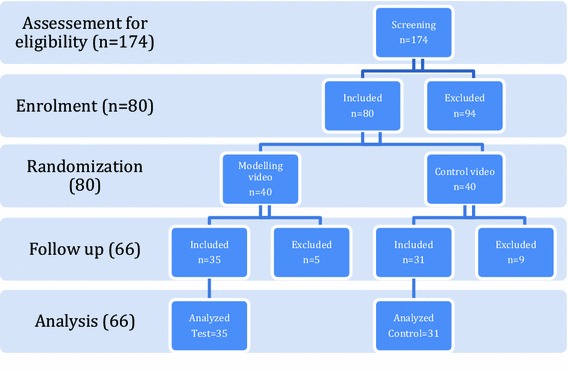
The flow chart of the participant throughout the randomised clinical trial to assess the influence of a modelling video on acceptance of a nasal mask for inhalation sedation

On the first visit, 80 children were randomly assigned to the modelling video (*n* = 40) or the control video (*n* = 40). On the second visit, five children from the modelling group were excluded, two failed to watch the video, three dropped out, and nine children from the control group were excluded (dropped out), but children who failed to watch the video from the control group were not excluded. Therefore, 66 children (35 modelling, 31 controls) had their results analysed. The flow chart of the participants is shown in Fig. 1. For both video groups, age was approximately normally distributed, the minimum age was 8 years and the maximum age was 16 years, the mean age was 12 years. The male and female participants were approximately equally distributed in the two groups: 55.2 % (16 out of 29) of the test group were males, and 44.8 % (13 out of 29) of the control group were males.

### Video modelling and dental anxiety scores

The total scores for the dental part of the ACDAS had a possible range of 13–39. The mean of the difference between the first visit ‘before watching the video’, and the second visit ‘after watching the video’ was calculated. There was a significant difference in the change in ACDAS score from the first to second visit between the test and control groups for the response on Question 12 on ACDAS ‘How do you feel about wearing a small rubbery mask on your nose to breathe special gas to help you feel comfortable during treatment?’ (*P* < 0.001), where the responses at each visit were coded 1, 2 and 3 for ‘Happy’, ‘OK’ and ‘Scared’, respectively. The total DA score difference before and after watching the video for the modelling group was 9.83, SD 4.99, and 0.26, SD 1.69 for the control group. In the modelling group 22.2 % of the participants showed no change in the DA scores for the nasal mask administration before and after watching the video, whereas 77.8 % of the control group showed no change in the DA score.

### Dental anxiety scores on VAS

**Fig. 2:**
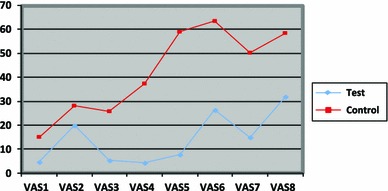
The mean of VAS at each of 8 stages for the modelling and control groups

The score of DA was reported by each child throughout the dental treatment stages, the data are summarised in Table 1. The summary of the VAS scores throughout the eight clinical stages for test and control groups is shown in Fig. 2, where the *Y* axis represents the VAS scores, and the *X* axis represents the treatment stages (VAS1, sitting in the waiting area; VAS2, entering the dental clinic; VAS3, sitting on the dental chair; VAS4, dental examination with mirror; VAS5, nasal mask application for IS; VAS6, dental injection; VAS7, tooth drilling; and/or VAS8, tooth extraction).

**Table 1:** The level of dental anxiety throughout the treatment

VAS stages	Modelling group	Control group	*P* value	Difference in means
In the waiting room	Mean 4.66, SD 8.02	Mean 15.07, SD 18.27	0.003	−10.41 (95 % CI −17.21 to −3.61)
Entering the dental clinic	Mean was 19.88, SD 22.13	Mean 28.15, SD 21.24	0.13	−8.27 (95 % CI −18.96 to 2.43)
Sitting on the dental chair	Mean 5.32, SD 9.12	Mean 25.81, SD 21.24	0.001	−20.48 (95 % CI −28.36 to −12.61)
Examination with mirror	Mean 4.34, SD 10.81	Mean 37.35, SD 25.43	*P* < 0.001	−33.01 (95 % CI −42.45 to −23.56)
Nasal mask application	Mean 7.79, SD 15.24	Mean 59.04, SD 30.93	*P* < 0.001	−51.25 (95 % CI −62.86 to −39.63)
Local anaesthesia	Mean 26.34, SD 26.01	Mean 63.5, SD 30.35	*P* < 0.001	−37.16 (95 % CI −51.02 to −23.3)
Tooth drilling	Mean 14.95, SD 24.83	Mean 50.25, SD 22.73	*P* < 0.001	−35.30 (95 % CI −50.28 to −20.32)
Tooth extraction	Mean 31.92, SD 30.53	Mean 58.47, SD 28.19	*P* = 0.004	−26.54 (95 % CI −44.23 to −8.85)

### Study outcomes

The primary outcome for this study was the change in the score of dental anxiety on the ACDAS scale from the first to the second visit. The secondary outcome was the score of the dental anxiety on the VAS of nasal mask application.

## Discussion

Although there were attempts to investigate the effect of video modelling on dental anxiety by previous studies (Melamed et al. 1975, 1978; Thelen et al. 1979), to date there is no RCT to investigate the effect of video modelling on the behaviour of anxious children receiving dental treatment. Therefore, this study is the first RCT for the use of video modelling in paediatric dentistry. Appropriate numbers, as determined by statistical evaluation, were collected in both study groups ensuring that the optimal sample size was achieved. Therefore, results from this study can be treated with some confidence.

To minimise potential bias in data collection, it is strongly recommended that the person gathering the study data and the clinician treating the patient should not be the same person (Streiner and Norman 2008). Therefore, an independent operator performed the clinical treatment. The data collection was completed by the chief investigator (AA).

In general, the test group was more successful at treating dental anxiety and at alleviating fear of the unknown (*P* < 0.001) than the control group, presumably because the test group was aware of what was going to happen to them after watching the modelling video. In the response of the key question (Q.12 of ACDAS): How do you feel about wearing a small rubbery mask on your nose to breathe special gas to help you feel comfortable during treatment?), 22.2 % (8 out of 36) for the test group and 77.8 % (28 out of 36) for the control group showed no change in the response for acceptance of the nasal mask application after watching the modelling video. On the VAS, this difference was also significant between modelling and control groups (*P* < 0.001). Hence, the video modelling was able to decrease the anxiety scores at the time of the nasal mask application for the modelling group.

Generally, there was a significant difference between modelling and control groups in the mean VAS throughout the rest of the dental treatment (*P* < 0.001). In the waiting room, the dental anxiety for both groups was very low, perhaps because EDH has a very child-friendly waiting area with many activities and entertainments for children. The level of DA was slightly raised when a child entered the dental clinic from the waiting room, although there was no significant difference in the mean VAS between the modelling and control groups (*P* = 19.88). At the time when any of the children sat on the dental chair, the DA level seemed to decrease on average, and to be even less at the time of the dental examination with the mirror.

The percentage of the GA referral was very high being 91.5 % for the children who were eligible to participate in this study, the decision of the GA referrals was always done by a senior specialist in paediatric dentistry. The video modelling method was chosen over the GA if this was possible by 98.3 % of the participants. Clearly patients and their caregivers would like to avoid GA if possible; however, it was outside the scope of this study to look at reasons for selection of GA. Obviously, however, alternatives to GA should always be offered wherever possible. The recruitment for this study was difficult at times as staff within the department would refer patients for GA frequently. In the absence of rigid criteria for referral to GA, this is something that perhaps should be the subject of future research.

The aim of the study was to investigate if use of a video film would decrease anxiety surrounding delivery of dental treatment facilitated by IS. Again whilst it was clear that use of the video film reduced anxiety surrounding nasal mask application, it was difficult to show if it would increase uptake. It is needed to determine if anxiety over dental treatment following this procedure is likely to decrease as a result of the modelling video film intervention.

A previously published systematic review found that the quality of reporting of clinical trials was poor, and often not adequate to allow readers to assess trial validity (Al-Namankany et al. 2009). Therefore, the reporting of this clinical trial conforms to the CONSORT statement.

The limitation of this study was that the child in the video film was not similar in terms of age, gender and ethnic background to every patient in the study, the possibility of the effect of these factors on the modelling outcomes is recommended for future research. In addition, modelling the child’s demographic was not feasible in terms of practicalities and costs. Furthermore, it was not feasible to provide a video model for every different dental treatment; the idea of the video was to give the child a general idea about the basics and the most commonly used procedures in the dental clinic.

In general, participants felt that the video was of a high quality. What could not be quantified was how important the video film quality was in terms of obtaining valid and useful results. This could be the subject of future research.

## Conclusions

Video film modelling appeared to be effective at reducing dental anxiety and had a significant impact on the acceptance of the nasal mask administration for inhalation sedation in children. All videos films are available to share, by contacting the first author.
